# A Survey on the Electronic Discharge Summary Process in an Acute Inpatient Ward

**DOI:** 10.1192/bjo.2024.527

**Published:** 2024-08-01

**Authors:** Ruth Akani

**Affiliations:** South West Yorkshire Partnership NHS Foundation Trust, Halifax, United Kingdom

## Abstract

**Aims:**

The study aimed to assess staff understanding of the discharge process in an Elmdale ward, Halifax and improve the promptness of discharge reports to other primary care professionals.

**Methods:**

Initially, the discharge process was reviewed in March 2023 to establish a baseline, focusing on completion time and personnel involved in the process. An online survey was conducted using Survey Monkey with 20 responses from the staff, including nurses, pharmacists, and doctors, to gather insights into their comprehension of the discharge process.

Electronic data for EPMA (electronic prescribing and medication administration) discharge form from SystmOne was analyzed to determine the percentage of completed discharge summaries and identify any incomplete or absent summaries among patients discharged from Elmdale ward (an acute inpatient ward) between March 1st and March 31st, 2023.

**Results:**

The data showed that 76.9% of discharges were completed within 24 hours, with weekend discharge completion at 4 and only 25% after 5 pm. Half of the discharge summaries were closed by nurses, 46% by doctors, and one by the ward clerk.

The median time taken to complete the discharge process was 25.83 hours, slightly exceeding the 24-hour target. Survey results indicated that 60% of staff were aware of the 24-hour timeline, but there were gaps in communication between staff members. Additionally, only 40% of staff had received formal EPMA discharge summary training, with nursing staff being the majority.

Eighty percent of survey respondents expressed challenges with the discharge summary process, particularly regarding communication with the pharmacy team and closing the discharge summary. Weekend discharge data revealed gaps in responsibilities when the ward clerk was unavailable to send letters.

Overall, the findings suggest a need for improved communication and training to enhance the efficiency and effectiveness of the discharge process, ensuring timely and accurate transmission of discharge reports to primary care physicians and other professionals.

**Conclusion:**

More than half of the staff understood the discharge process however communication between staff in regard to the discharge process impacted on the timeliness of the summaries completed.

